# The efficacy of ‘static’ training interventions for improving indices of cardiorespiratory fitness in premenopausal females

**DOI:** 10.1007/s00421-018-4054-1

**Published:** 2018-12-27

**Authors:** P. J. J. Herrod, J. E. M. Blackwell, B. F. Moss, A. Gates, P. J. Atherton, J. N. Lund, J. P. Williams, B. E. Phillips

**Affiliations:** 1 MRC-ARUK Centre for Musculoskeletal Ageing Research, University of Nottingham, Royal Derby Hospital Centre, DE22 3DT Derby, UK; 2 Department of Anaesthetics and Surgery, Royal Derby Hospital, Derby, UK

**Keywords:** Ischaemic preconditioning, Isometric handgrip, Cardiovascular risk, Interventions

## Abstract

**Purpose:**

Cardiovascular disease (CVD) is the leading cause of death worldwide. Many risk factors for CVD can be modified pharmacologically; however, uptake of medications is low, especially in asymptomatic people. Exercise is also effective at reducing CVD risk, but adoption is poor with time-commitment and cost cited as key reasons for this. Repeated remote ischaemic preconditioning (RIPC) and isometric handgrip (IHG) training are both inexpensive, time-efficient interventions which have shown some promise in reducing blood pressure (BP) and improving markers of cardiovascular health and fitness. However, few studies have investigated the effectiveness of these interventions in premenopausal women.

**Method:**

Thirty healthy females were recruited to twelve supervised sessions of either RIPC or IHG over 4 weeks, or acted as non-intervention controls (CON). BP measurements, flow-mediated dilatation (FMD) and cardiopulmonary exercise tests (CPET) were performed at baseline and after the intervention period.

**Results:**

IHG and RIPC were both well-tolerated with 100% adherence to all sessions. A statistically significant reduction in both systolic (− 7.2 mmHg) and diastolic (− 6 mmHg) BP was demonstrated following IHG, with no change following RIPC. No statistically significant improvements were observed in FMD or CPET parameters in any group.

**Conclusions:**

IHG is an inexpensive and well-tolerated intervention which may improve BP; a key risk factor for CVD. Conversely, our single arm RIPC protocol, despite being similarly well-tolerated, did not elicit improvements in any cardiorespiratory parameters in our chosen population.

## Introduction

Cardiovascular disease (CVD) represents a significant global health burden, being the leading cause of death worldwide (GBD 2013 Mortality and Causes of Death Collaborators 2015). To exemplify the modifiable nature of CVD risk, known risk factors such as hypertension, dyslipidaemia, smoking, diabetes, abdominal obesity, diet and physical inactivity may all be influenced and have been held accountable for over 90% of the risk of having an acute myocardial infarction (Bonow 2002; Yusuf et al. 2004). A number of these risk factors may be modified using medication, however, they are not without side effects (Finegold et al. 2014; Taylor and Thompson 2015) and compliance remains poor (Peterson and McGhan 2005; Gislason et al. 2006; Newby et al. 2006; Larbizabal and Deedwania 2010). In addition, patients are often unwilling to take medications in the absence of symptoms (Ross et al. 2004; Munger et al. 2007).

Non-pharmacological interventions (i.e., diet and exercise) can also modulate CVD risk (Hellenius et al. 1993; Bassuk and Manson 2005). However, despite these well-publicised benefits, the uptake of physical activity remains low. Indeed, one-third of adults and four-fifths of adolescents worldwide fail to meet physical activity guidelines (Hallal et al. 2012). Exercise referral schemes have long been promoted as a means of increasing physical activity (Fox et al. 1997), but have variable compliance and efficacy (Dugdill et al. 2005; Williams et al. 2007). Key cited barriers to physical activity include financial cost, childcare access and crucially, lack of time (Withall et al. 2011). These barriers make the prospect of an inexpensive, time-efficient form of physical conditioning, which can be performed at home without specialist equipment very attractive.

Repeated remote ischaemic preconditioning (RIPC) (Epps et al. 2016) and isometric handgrip training (IHG) (Inder et al. 2016) are two simple interventions which have been shown to have the potential to modify CVD risk factors. RIPC involves repeatedly alternating periods of ischaemia and reperfusion in a muscle bed in an attempt to precondition the body to withstand future periods of hypoxia. IHG involves sustained repeated contractions of the hand around a dynamometer maintaining a fixed muscle length. Single session RIPC performed immediately prior to exercise has been shown to improve athletic performance in both competitive athletes (Jean-St-Michel et al. 2011) and recreationally active young individuals (Cruz et al. 2015, 2016). However, the few studies of repeated RIPC that exist (over 7 days to 8 weeks) have had mixed effects on cardiorespiratory fitness (CRF) (Jones et al. 2014; Banks et al. 2016; Lindsay et al. 2017), a validated predictor of future CVD risk (Kodama et al. 2009). It must, however, be noted that the majority of these studies were in male volunteers. The potential mechanisms behind how repeated RIPC could mediate improvements in CRF are still unknown, with local effects on the muscle being preconditioned as well as remote effects via both neural pathways and humoral factors being postulated (Sharma et al. 2015). Several studies have reported the effects of repeated RIPC on resting blood pressure (BP), although as yet a consensus on its efficacy has not been reached (Epps et al. 2016). Conversely, IHG has been shown in a number of trials to reduce resting BP in both normotensive and hypertensive individuals (Inder et al. 2016). However, the only two studies to date which measured changes in CRF after a prolonged period of IHG did not show benefit (Blackwell et al. 2017; Goessler et al. 2018), moreover, the larger of these two studies was of unsupervised IHG with a reported adherence as low as 63%.

Another predictor of CVD risk (alongside BP and CRF) is endothelial dysfunction (Bonetti et al. 2002). Reduced endothelial function may precede overt vascular disease by a number of years (Anderson et al. 1995) and can be estimated by brachial artery ultrasound scanning to detect flow mediated dilatation (FMD) in response to shear stress applied to the artery wall (Faulx et al. 2003; Pyke and Tschakovsky 2005; Charakida et al. 2010). This measure of endothelial plasticity is commonly used as a surrogate for cardiovascular health and has been validated as a means of assessing vascular responsiveness (Charakida et al. 2010). IHG has been shown to improve FMD in medicated hypertensive (McGowan et al. 2007b), but not normotensive subjects (McGowan et al. 2007a), over an 8-week period. Similarly, RIPC has shown promise with improvements in FMD demonstrated after 2-week intervention (Jones et al. 2014) in a young normotensive healthy population. However, as with the aforementioned studies assessing the effects of these ‘static’ interventions on CRF and resting BP, almost all of the study subjects were male.

The current paucity of any substantial studies investigating these interventions in premenopausal females means no firm conclusions can yet be drawn as to whether they have any role to play in CVD risk modulation in this population. This is of increasing importance with many traditional CVD risk factors now increasing in this population (Garcia et al. 2016). This study was therefore designed to investigate the efficacy of 4 weeks supervised RIPC or IHG on CRF, BP and endothelial function in inactive but otherwise healthy, premenopausal females. A 4-week intervention was chosen to try to minimise natural variation in physiological parameters within subjects due to phases of their menstrual cycle (Mihm et al. 2011). We hypothesised that both RIPC and IHG would each improve at least one parameter associated with CVD risk.

## Methods

### Subject characteristics

Institutional research ethics approval (University of Nottingham Medical School Ethics Committee) was obtained (J14112013 SoM MS GEM) before healthy (non-hypertensive) inactive female subjects aged 25–50 years 37.9 (7.9); BMI > 18 or < 30 kg/m^2^: [23.8 (3.1)] were recruited by local advertising. Activity status was defined as no participation in any formal exercise regime. After obtaining written informed consent, 30 subjects were recruited to this study. Twenty were randomised to either IHG or RIPC, with ten subjects allocated to a non-intervention control group. There were no significant differences in any baseline demographic characteristics between the groups (Table 1). All methods were performed in accordance with the relevant guidelines and regulations. The study was registered with clinicaltrials.gov on 31 October 2017 (NCT03473990) and complied with the 1964 Declaration of Helsinki.

**Table 1:** Subject characteristics

Group	Age (years)	Weight (kg)	Height (m)	BMI (kg/m^2^)
RIPC	38 (7)	66.1 (9.3)	1.68 (0.07)	23.4 (2.9)
IHG	33 (9)	63.0 (10.3)	1.65 (0.07)	23.2 (3.6)
Control	38 (7)	66.0 (7.0)	1.62 (0.05)	25.0 (2.0)
*p* value	0.34	0.69	0.20	0.33

*p* value shows main effect of one-way ANOVA across groups for each parameter

*RIPC* Remote ischaemic preconditioning, *IHG* isometric handgrip training

Before baseline testing, all subjects underwent cardiovascular examination by a qualified medical doctor with exclusion criteria for further participation as per the ATS/ACCP guidelines for Cardiopulmonary Exercise Testing (CPET) (Weisman et al. 2003). At baseline testing, participants underwent measurements of resting BP and FMD and completed a CPET. After the 4-week intervention (or control) period, the same tests were repeated.

### Pre- and post-intervention testing

For both testing sessions, subjects attended the laboratory fasted from midnight at approximately 0900 h. Supine BP was measured in triplicate after a 5-min supine resting period using oscillometry (Datascope trio patient monitor, Datascope, New Jersey, USA) and a blood pressure cuff (Welch Allyn, New York, USA) of appropriate size (British and Irish Hypertension society 2017) using the subject’s right arm.

After a further 30-min supine in a 24 °C temperature-controlled room, FMD was assessed according to the International Brachial Artery Reactivity Task Force guidelines (Corretti et al. 2002) in the subject’s right arm. In brief, after a baseline measurement of brachial artery diameter for 1-min, arterial occlusion distal to the brachial artery was induced using a BP cuff (Hokanson, Washington, USA) inflated to 200 mmHg for 5-min. The cuff was then deflated and dilatation of the brachial artery assessed for a further 5-min. A linear array ultrasound transducer (17 − 5 MHz with Philips iU22 ultrasound machine; Philips Healthcare, Amsterdam, Netherlands) was used for the FMD imaging, with automated real-time arterial diameter measurements (Quipu Cardiovascular Suite FMD Studio version 3.2.0; Quipu, Pisa, Tuscany, March 2017) (Gemignani et al. 2008).

CPET was then performed according to ATS/ACCP guidelines (Weisman et al. 2003) using a Lode Corival cycle ergometer (Lode Corival, Lode, Groningen) and inline gas analysis system (ZAN 680, nSpire Health, Colorado, USA). After 2-min of unloaded cycling, participants were instructed to maintain a cadence of 50–60 revolutions per minute while being encouraged to exercise to volitional exhaustion. A Bruce ramp protocol (Kaminsky and Whaley 1998; Will and Walter 1999) was selected (10–25 W/min) based on the participant’s body weight and self-reported level of habitual physical activity to ensure the CPET was between 8- and 12-min in duration (Buchfuhrer et al. 1983; Weisman et al. 2003). Anaerobic threshold (AT) was determined using a combination of the V-slope and VE methods (Wasserman et al. 1973; Beaver et al. 1986) by two blinded independent assessors, with disagreement resolved by consensus.

The first RIPC or IHG training session was carried out within 7-days of pre-intervention testing and the post-intervention tests carried out within 2–5 days of the final training session. This time interval duration was chosen so as to avoid the potential acute effect of the interventions on blood pressure (Farah et al. 2017).

### Interventions

Subjects assigned to RIPC or IHG attended the laboratory three times each week for 4-weeks, with each session lasting approximately 15-min. All sessions were fully supervised by a member of the research team. Control subjects attended only for pre- and post-intervention testing sessions. All subjects were asked to maintain their habitual level of physical activity for the duration of the study.

**Fig. 1:**
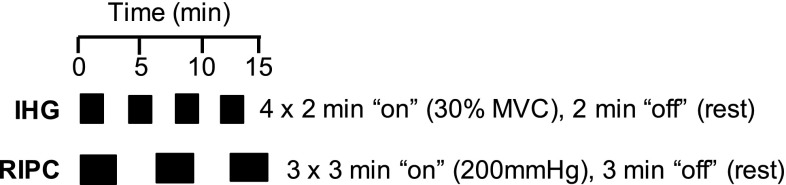
Schematic representation of isometric handgrip training (IHG) and remote ischaemic preconditioning (RIPC) intervention protocols, each performed 3 ×/week for 4-weeks

RIPC subjects were conditioned with a manual non-invasive blood pressure cuff (Welch Allyn, NY, USA). At each session, this cuff was placed on their right arm and inflated to 200 mmHg for 3-min before deflating. This was repeated three times in each session with 3 min rest between each inflation (Fig. 1). The decision to use a single, upper-limb protocol was taken based on this being a protocol employed by a number of studies in the most recent systematic review (Epps et al. 2016). Limb occlusion time in this study was less than in many previously studied protocols, to reduce the total time commitment for the training protocol, making it comparable with the IHG intervention.

At each session, subjects assigned to IHG completed four 2-min repetitions of isometric handgrip at 30% of maximum voluntary contraction (MVC) on an electronic handgrip dynamometer (Camry EH101, Zhongshan Camry Electronic Co. Ltd, Guangdong, China) using their right arm, with 2 min rest between each contraction (Fig. 1). Handgrip MVC was assessed at the first IHG session using the same dynamometer as used for IHG, whilst subjects were seated with elbow flexion of 90° [as per the American Society of Hand Therapist recommendations (Fess 1992)]. The maximum from three repetitions of maximum effort (allowing 1-min rest between attempts) was recorded as MVC.

### Statistics

All calculations were performed using Graphpad Prism Version 7.02 (California, USA). Data are presented as mean (SD). Participant demographics at baseline were compared using one-way ANOVA, whilst outcome data were compared using two-way ANOVA both with Tukey’s post hoc analysis. Significance was taken as an alpha of *p* < 0.05.

## Results

### Feasibility

All participants completed the 4-week study with none lost to follow-up. All training was directly observed, and both interventions were well tolerated, with compliance 100%, including baseline testing and re-assessment. There were no adverse events throughout the study (as defined by Good Clinical Practice) (ICH 2016) and verbal feedback from all participants indicated a high acceptability of the interventions.

### Resting blood pressure

**Fig. 2:**
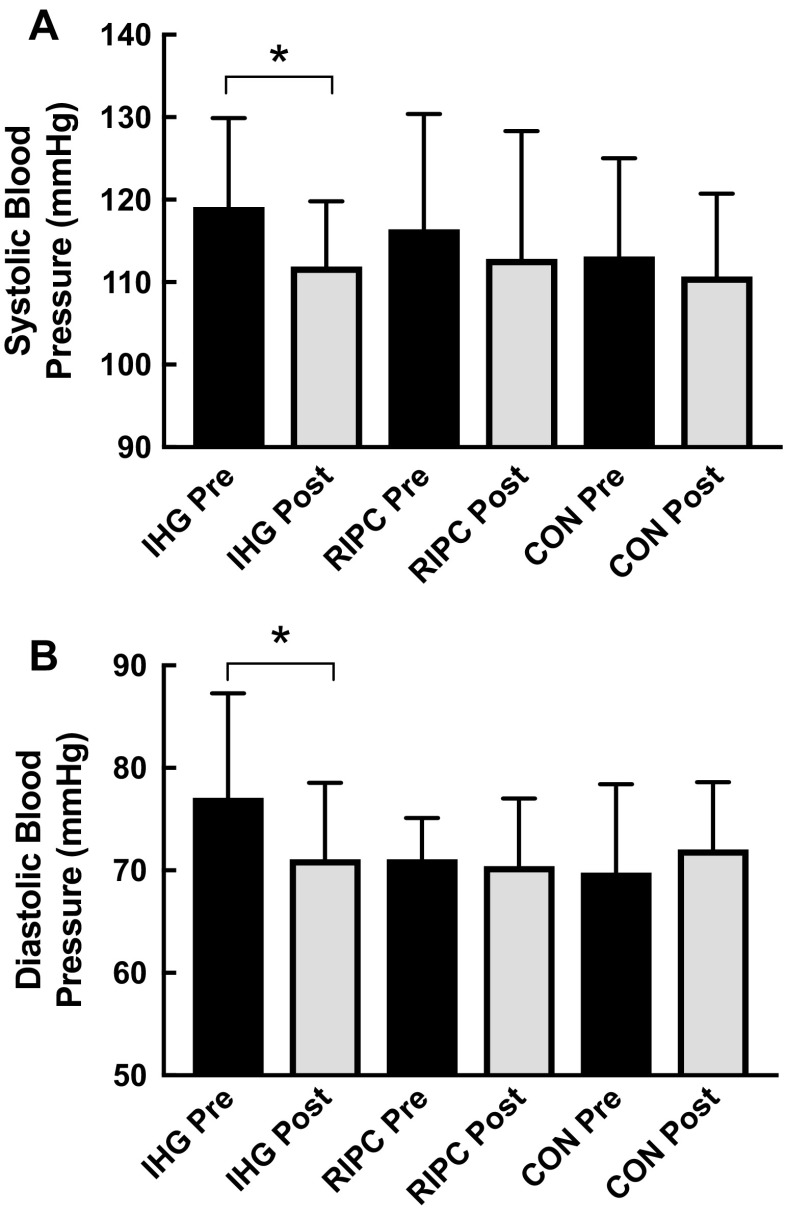
Systolic (**a**) and diastolic (**b**) blood pressure before and after 4-weeks remote ischaemic preconditioning (RIPC), isometric handgrip training (IHG) or a control period. Data are presented as Mean +/- SD. *N* = 10 subjects per group

There was no statistically significant difference in either systolic blood pressure (SBP) or diastolic blood pressure (DBP) between any of the groups at baseline. There was a statistically significant reduction in SBP in the IHG group after training [Pre: 119.1 (10.8) mmHg vs. Post: 111.9 (7.9) mmHg, *p* = 0.03, mean reduction: 7.2 (10.6) mmHg], but no significant change in either the RIPC [Pre: 116.4 (14.0) mmHg vs. Post: 112.8 (15.5) mmHg, *p* = 0.46] or the control [Pre: 113.1 (11.9) mmHg vs. 110.7 (10.0) mmHg, *p* = 0.75] groups (Fig. 2a). There was a main effect of time on SBP (*p* = 0.01) across all groups.

There was a statistically significant reduction in DBP in the IHG group after training [Pre: 77.1 (10.2) mmHg vs. Post: 71.1 (7.4) mmHg, *p* = 0.03, mean reduction: 6.0 (9.4)mmHg], but no significant change in either the RIPC [Pre: 71.1 (4.0) mmHg vs. 70.4 (6.6) mmHg, *p* = 0.98] or the control [(Pre: 69.8 (8.6) mmHg vs. 72.0 (6.6) mmHg, *p* = 0.69] groups (Fig. 2b). There was no main effect of time on DBP (*p* = 0.3) across all groups.

### Cardiopulmonary exercise testing

As was the case for blood pressure values, there were no statistically significant differences in CRF between the groups at baseline. There was no significant change in the CPET-derived parameters of AT, VO_2_ peak or peak workload in any group (Table 2), with no main effect of time across the groups for any CPET variable (all *p* = 0.3).

**Table 2:** Cardiorespiratory fitness before and after 4-week remote ischaemic preconditioning (RIPC), isometric handgrip training (IHG) or a control period

	AT pre	AT post	AT change	*p* value	VO_2_peak pre	VO_2_peak post	VO_2_peak change	*p* value	Power pre	Power post	Power change	*p* value
RIPC	15.8 (3.4)	16.8 (4.4)	1.1 (1.4)	0.43	32.8 (7.3)	33.7 (5.7)	0.8 (2.1)	0.74	184 (35)	188 (33)	4 (8)	0.90
IHG	19.0 (5.3)	18.7 (5.0)	− 0.3 (3.2)	0.98	38.7 (7.2)	36.5 (6.9)	− 2.2 (3.3)	0.06	203 (30)	215 (35)	13 (33)	0.16
Control	15.9 (2.5)	16.4 (3.8)	0.4 (2.3)	0.91	35.2 (7.9)	34.9 (8.6)	− 0.3 (3.0)	0.97	192 (36)	187 (40)	− 5 (10)	0.83

*AT* Volume of oxygen uptake at anaerobic threshold (ml/kg/min), *VO*_*2*_*Peak* peak volume of oxygen uptake during a cardiopulmonary exercise test (ml/kg/min), *Power* peak power during a cardiopulmonary exercise test (Watts)

### Endothelial function

As with the other outcomes, there were no statistically significant differences in FMD at baseline between the groups. There was no significant change in FMD, our marker of endothelial function, after intervention in any group [IHG: 6.9 (3.9) vs. 8.0 (2.6)%, + 1.1 (4.5)%, *p* = 0.81; RIPC: 5.9 (3.9) vs. 6.9 (6.1)%, + 1.0 (4.7)%, *p* = 0.84; CON: 5.8 (2.5) vs. 6.1 (3.4)%, + 0.3 (2.9)%, *p* = 0.99), with no main effect of time (*p* = 0.3) across the groups.

## Discussion

This study is the first to compare the effects of IHG and RIPC on markers of cardiovascular health in inactive otherwise healthy premenopausal females. It demonstrates that IHG and RIPC are both well-tolerated in this group, with 100% compliance and no adverse events. We showed that IHG, conducted over 4 weeks can lead to statistically significant reductions in both SBP and DBP but was unable to elicit any change in CRF. We did not detect changes in any measures of cardiovascular health or fitness from our RIPC protocol, questioning the application of this particular time-efficient RIPC protocol to reduce CVD risk in this specific population.

The significant reduction in SBP in our IHG group mirrors the findings of Gill et al. who observed a 6.9-mmHg reduction in SBP in normotensive females after 3-weeks of isometric exercise training (IET) (Gill et al. 2015). However, Gill et al. employed bilateral leg extensions as their mode of IET, thus recruiting a much larger muscle. We have demonstrated a similar reduction in BP using only a handgrip protocol, which may have greater potential for translation to real-world use. Of note, a previous meta-analysis has demonstrated that IET reduces blood pressure most effectively in hypertensive males (Inder et al. 2016), as the subjects in this study were neither hypertensive nor male the potential for greater improvements in other cohorts with IHG remains.

The absence of a significant change in SBP and DBP in our RIPC group is in keeping with a previous study of RIPC in young adults which also showed no change in BP or exercise capacity in normotensive male and female participants (Banks et al. 2016).

We did not demonstrate significant improvements in any CPET parameter in any group. This mirrors the findings of the only previous study assessing CPET variables before and after a prolonged period of IHG (Blackwell et al. 2017). Similarly, this finding also agrees with two previous studies that have performed a CPET before and after repeated RIPC (Jones et al. 2014; Banks et al. 2016). It is noteworthy that despite a lack of improvement in CPET parameters with repeated RIPC, a single-bout of RIPC performed immediately prior to exercise has been shown to improve athletic performance (Jean-St-Michel et al. 2011; Cruz et al. 2015). This suggests that the mechanisms by which a single-bout of RIPC improve athletic performance [postulated to be either through improved metabolic efficiency or increased limb blood flow (Incognito et al. 2016)] are not retained over the long-term and cannot be further improved and/or maintained by repeated sessions.

This study also failed to demonstrate any significant improvement in FMD in any group. This is in keeping with previous studies that have shown IHG to be effective at increasing FMD in hypertensive subjects only (McGowan et al. 2007a, b). However, the only previous study investigating the effect of RIPC on FMD (Jones et al. 2014) did demonstrate a significant improvement in healthy normotensive young males, this is contrary to the findings reported herein. The lack of effect of RIPC observed in this study may indicate sexual dimorphism in the effects of RIPC on FMD, a postulation that has also been suggested for other cardiovascular parameters in response to static interventions (Gill et al. 2015).

One limitation of this study was the inability to control for the length of the menstrual cycle of the subjects or their stage of menstrual cycle whilst participating in the study. Stage of menstrual cycle is known to effect physiological parameters and this may have confounded our results (Dunne et al. 1991). However, recruiting a group of subjects with identical menstrual cycles and/or running the testing days on a set-day of the cycle would not have been feasible in our institution.

It must also be acknowledged that our single arm, 3 × 3-min of ischaemia protocol for our RIPC intervention involved less ischaemic time that many other previous studies of RIPC (Banks et al. 2016, Epps et al. 2016). We chose this protocol to limit the time commitment of training sessions, making it comparable to the IHG intervention, however, in doing so we may have not delivered a sufficient ischaemic stimulus. Future studies of RIPC should consider using a longer occlusion time ischaemic occlusion of a larger muscle mass.

Based on the findings of this study and the previous literature in this area, IHG, deliverable through a single-arm handgrip protocol may lead to significant reductions in resting blood pressure in premenopausal women, while repeated RIPC using our single arm, 3-min ischaemia protocol appears to be ineffective in this population. Further work is required to determine the effects of RIPC and IHG on physiological parameters associated with CVD risk in female subgroups in which this risk is elevated (e.g., hypertensives, older females). Given the tolerability of these simple, inexpensive interventions as shown in this study, if proven effective, these interventions may offer large public health benefit as independent or adjuvant [i.e., alongside established exercise and dietary changes (Warburton et al. 2006; Cook et al. 2007)] lifestyle recommendations to improve cardiovascular health in specific groups.

## Abbreviations

ANOVA: Analysis of variance
AT: Anaerobic threshold
BMI: Body mass index
BP: Blood pressure
CON: Non-intervention control group
CPET: Cardiopulmonary exercise test
CRF: Cardiorespiratory fitness
CVD: Cardiovascular disease
DBP: Diastolic blood pressure
FMD: Flow-mediated dilatation
IET: Isometric exercise training
IHG: Isometric handgrip training
MVC: Maximum voluntary contraction
RIPC: Remote ischaemic preconditioning
SBP: Systolic blood pressure
SD: Standard deviation
VO_2_ peak: Peak volume of oxygen uptake
